# Knowledge and perceptions of antimicrobial resistance and antimicrobial stewardship among staff at a national cancer referral center in Uganda

**DOI:** 10.1017/ash.2022.28

**Published:** 2022-04-06

**Authors:** Elizabeth A. Gulleen, Margaret Lubwama, Alfred Komakech, Elizabeth M. Krantz, Catherine Liu, Warren Phipps

**Affiliations:** 1 Vaccine and Infectious Diseases Division, Fred Hutchinson Cancer Research Center, Seattle, Washington, United States; 2 Allergy and Infectious Diseases Division, Department of Medicine, University of Washington, Seattle, Washington, United States; 3 Seattle Cancer Care Alliance, Seattle, Washington, United States; 4 Department of Medical Microbiology, Makerere University, Kampala, Uganda; 5 Department of Pharmacy, Uganda Cancer Institute, Kampala, Uganda

## Abstract

**Objectives:**

As access to cancer care has improved throughout sub-Saharan Africa, treatment-associated infections have increased. Assessing healthcare worker knowledge of antimicrobial stewardship and identifying the barriers to infection management will inform the development of contextually appropriate antimicrobial stewardship programs, improving cancer outcomes in sub-Saharan Africa.

**Design:**

Cross-sectional survey.

**Setting:**

The Uganda Cancer Institute (UCI), a national cancer referral center in Kampala, Uganda.

**Participants:**

We surveyed 61 UCI staff: 29 nurses, 7 pharmacists, and 25 physicians.

**Methods:**

The survey contained 25 questions and 1 ranking exercise. We examined differences in responses by staff role.

**Results:**

All 60 respondents who answered the question had heard the term “antimicrobial resistance.” Only 44 (73%) had heard the term “antimicrobial stewardship.” Nurses were less likely than pharmacists or physicians to be familiar with either term. Also, 41 respondents (68%) felt that loss of antibiotic susceptibility is a major issue at UCI. Regarding barriers to diagnosing infections, 54 (93%) of 58 thought that it was difficult to obtain blood cultures and 48 (86%) of 56 thought that it was difficult to regularly measure temperatures.

**Conclusions:**

Although most recognized the term “antimicrobial resistance,” fewer were familiar with the term “antimicrobial stewardship.” Inappropriate antibiotic use was recognized as a contributor to antimicrobial resistance, but hand hygiene was underrecognized as a contributing factor. We identified numerous barriers to diagnosing infections, including the ability to obtain blood cultures and consistently monitor temperatures. Educating staff regarding antimicrobial selection, allocating resources for blood cultures, and implementing strategies to enhance fever detection will improve infection management.

By 2030, 1.28 million new cancer cases and 970,000 cancer-related deaths are anticipated in sub-Saharan Africa annually.^1^ Over the past 20 years, access to cancer diagnosis and treatment has expanded; however, improved access to treatment has resulted in increased treatment-related infections.^2–4^ In sub-Saharan Africa, the growing prevalence of multidrug-resistant (MDR) bacterial infections has emerged as a public health emergency.^5,6^ Those with cancer are at increased risk of developoing MDR bacterial infections due to frequent healthcare exposure and high rates of antibiotic use. This is particularly concerning because MDR bacterial bloodstream infections are associated with high mortality among those with cancer.^7^ In sub-Saharan Africa, limited access to microbiology laboratories and the high cost of blood cultures make it difficult to identify patients with MDR bacterial infections.^8^ When identified, these patients often require treatment with second-line antibiotics that are expensive and may not be readily available. Because there are relatively few trained infectious diseases specialists in sub-Saharan Africa,^9^ it is important to ensure that all members of the clinical oncology team understand the high prevalence of MDR bacterial infections and are well versed in evidence-based antibiotic management practices.

Antimicrobial stewardship is a coordinated group of interventions designed to improve appropriate antimicrobial use by optimizing treatment regimens.^10^ The World Health Organization developed a practical tool kit to guide the design and implementation of antimicrobial stewardship programs (ASPs) in low-resource settings.^10^ However, patients with cancer are a vulnerable population with unique diagnostic and antimicrobial management needs.^11^ Many are immunosuppressed due to their underlying disease or cancer treatment regimens. In sub-Saharan Africa, up to 35% of those with cancer also have human immunodeficiency virus (HIV), which is associated with increased rates of antimicrobial resistance. Although ASPs have been developed for patients receiving cancer treatment in high-resource settings,^12^ they have not been adapted to account for the high prevalence of MDR bacteria, resource limitations, and local healthcare infrastructure in lower- and middle-income countries (LMICs). Because patients receiving cancer treatment in sub-Saharan Africa have high rates of infection-related mortality,^2,13^ implementing locally adapted ASPs could significantly improve patient outcomes. To develop these programs, it is critical to assess healthcare workers’ baseline knowledge of antimicrobial resistance and antimicrobial stewardship and to explore the unique challenges faced when diagnosing and treating infections in a low-resource setting.

We evaluated the knowledge and attitudes regarding antimicrobial resistance and antimicrobial stewardship among physicians, nurses, and pharmacists at the Uganda Cancer Institute (UCI), a national cancer referral hospital in sub-Saharan Africa. We also assessed sources of antibiotic education and to understand the perceived barriers to infection diagnosis and treatment.

## Methods

### Study design, period, and setting

In April and May of 2021, we conducted a cross-sectional survey of UCI inpatient staff. The UCI is a national cancer referral center in Kampala, Uganda, and the East African Center of Excellence in Oncology. More than 5,000 adult and pediatric patients are treated annually in the 100-bed hospital and >20 ambulatory clinics. Patient care is provided by >169 staff, including 112 nurses, 9 pharmacists, and 48 physicians. Over the past 7 years, our group has collaborated on several infection management initiatives, and we have established a multidisciplinary antimicrobial stewardship team of microbiologists, infectious diseases physicians, pharmacists, oncologists, and nurses. Initiatives have included adapting international neutropenic fever guidelines to the local context and initiating an infectious diseases consultation service.

### Survey development

We designed a self-administered survey (1) to assess the knowledge, attitudes, and perceptions of antimicrobial resistance and antimicrobial stewardship, (2) to characterize current sources of antibiotic education, and (3) to identify perceived barriers to infection diagnosis and treatment (Supplementary File 1). The survey contained 26 questions and 1 ranking exercise. To create the survey, we used our knowledge of UCI clinical management practices and adapted questions from surveys designed to assess the unique challenges faced when diagnosing and treating infections in LMICs.^14^ We pilot-tested the survey on 10 US and Ugandan healthcare providers to optimize clarity and readability.

### Survey distribution

We compiled a list of staff who work on the UCI inpatient wards. We then used face-to-face conversations, phone calls, and texts messages to invite them to participate. Participants could choose to complete the survey on paper or online. For the paper survey, we gave the participants a copy, which they completed and returned to a designated study team member. For the online survey, we texted or e-mailed the participants a direct link to the REDCap survey. The completed surveys were entered into a REDCap database. The study team manually entered paper survey responses, while online survey responses were automatically uploaded.

### Data analysis

We tabulated survey responses, presented as frequencies and percentages. For each question, we excluded missing responses from analysis. We compared survey responses using nonparametric tests. To test for differences in binary variables for educational formats by staff role, we used the Fisher exact test for overall differences, followed by Fisher exact pairwise comparisons using the Holm method to adjust for multiple comparisons. To determine whether there were differences among any of the ordinal variables for knowledge regarding antimicrobial resistance and antimicrobial stewardship by staff role, we used the Kruskal-Wallis test. If significant, we used the Dunn test for pairwise comparisons between groups with the Holm adjustment for multiple comparisons to determine which variables differed significantly. We compared relative differences in responses across all Likert-type or ranking questions using the Friedman test. If significant, we performed pairwise comparisons using the exact test with the Holm adjustment to account for multiple comparisons to determine which questions differed significantly. We analyzed survey responses using R Studio software (R Foundation for Statistical Computing, Vienna, Austria) and Stata version 16.1 software (StataCorp, College Station, TX). We considered *P* < .05 statistically significant.

### Ethical considerations

The Fred Hutchinson Cancer Research Center Intitutional Review Board, the Uganda Cancer Institute Research and Ethics Committee, and the Uganda National Council on Science and Technology approved the study. We used a standardize script to obtain verbal consent and an anonymous study number to document consent.

## Results

### Understanding of antimicrobial resistance

Among the 75 staff who we identified as providing inpatient care, we were able to contact 65 (86%). Of these, 61 (94%) completed the survey (Table 1). Although all respondents had heard of the term “antimicrobial resistance” (Table 2), the survey revealed significant differences in degrees of knowledge based on staff role (*P* < .001). Nurses were less familiar with the term than pharmacists (*P* = .03) or physicians (*P* < .001).

**Table 1.** Baseline Demographics of Uganda Cancer Institute Staff Who Responded to the Antibiotic Survey Between April and May 2021

Characteristics	Total (n = 61), No. (%)
**Employee role**	
Nurse	29 (48)
Pharmacist	7 (11)
Physician	25 (41)
**Years in role**	
0–4	21 (34)
5–9	17 (28)
10–14	16 (26)
15+	7 (11)

**Table 2.** Participant Responses to Questions Regarding the Antimicrobial Resistance and Antimicrobial Stewardship By Staff Role

Survey Questions	All, No. (%)	Nurses, No. (%)	Pharmacists, No. (%)	Physicians, No. (%)
Total respondents	60^a^ (100)	28 (47)	7 (12)	25 (42)
**1. Have you heard of the term “antimicrobial resistance”?**				
I have not heard the term	0 (0)	0 (0)	0 (0)	0 (0)
I have heard the term, but do not know what it means	2 (3)	2 (7)	0 (0)	0 (0)
I have heard the term, and know a little about what it means	22 (37)	17 (59)	1 (14)	4 (17)
I have heard the term and know a lot about what it means	36 (60)	10 (34)	6 (86)	20 (83)
**2. Loss of antimicrobial sensitivity (development of antimicrobial resistance) is a major problem for patients at UCI.**				
Strongly disagree	2 (3)	1 (3)	0 (0)	1 (4)
Somewhat disagree	0 (0)	0 (0)	0 (0)	0 (0)
Neither agree nor disagree	1 (2)	1 (3)	0 (0)	0 (0)
Somewhat agree	17 (28)	11 (38)	1 (14)	5 (21)
Strongly agree	34 (57)	13 (45)	6 (86)	15 (62)
Don’t know	6 (10)	3 (10)	0 (0)	3 (12)
**3. Have you heard of the term “antimicrobial stewardship”?**				
I have not heard the term	16 (27)	12 (43)	0 (0)	4 (16)
I have heard the term, but do not know what it means	12 (20)	9 (32)	0 (0)	3 (12)
I have heard the term, and know a little about what it means	17 (28)	6 (21)	2 (29)	9 (36)
I have heard the term and know a lot about what it means	15 (25)	1 (3.6)	5 (71)	9 (36)

a Of the 61 respondents, 60 (98%) answered the questions regarding antimicrobial resistance and antimicrobial stewardship. One physician did not answer question 1 and 2 and 1 nurse did not answer question 3.

**Fig. 1.**
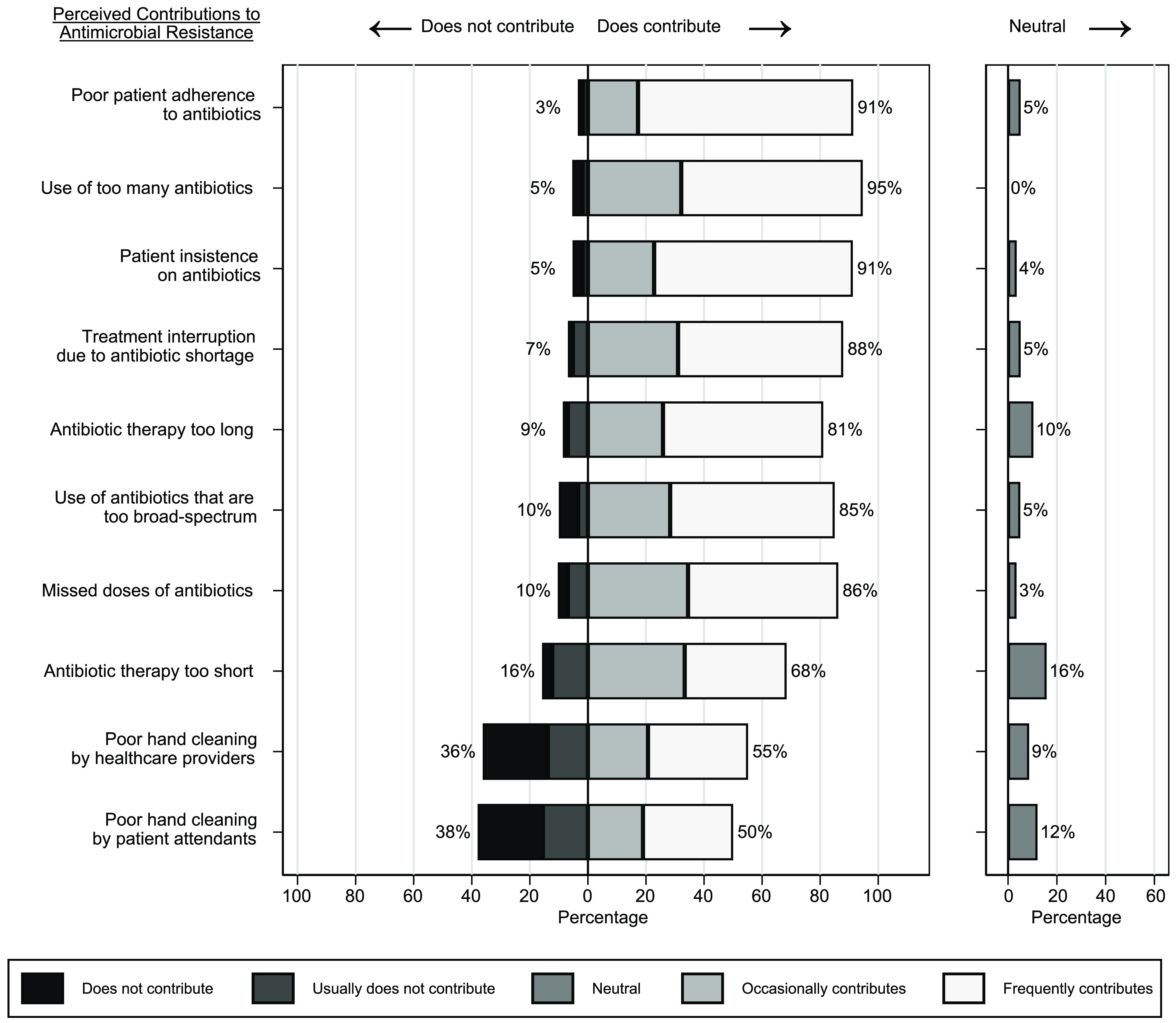
Factors that physicians, pharmacists, and nurses working at the Uganda Cancer Institute (UCI) perceive as contributing to antimicrobial resistance at the UCI. Percentages shown next to bars represent the combined total percentage of respondents reporting that the factor does not or usually does not contribute (left of bars, main chart), occasionally or frequently contributes (right of bars, main chart), or neither contributes nor does not contribute (right of neutral chart).

Most respondents agreed that loss of antibiotic susceptibility is a major problem at UCI (Table 2). We detected a significant difference in the degree to which various factors were felt to contribute to antimicrobial resistance (*P* < .001) (Fig. 1). Poor patient adherence to antibiotics, patient request for antibiotics (ie, insistence that antibiotics be prescribed), and the use of too many antibiotics were the most commonly identified contributing factors. Provider hand hygiene and attendant hand hygiene were the contributing factors least often identified. Overall, 56 (93%) of 60 respondents knew that giving antibiotics to a patient who does not have an infection can cause loss of antibiotic susceptibility. In addition, 39 respondents (65%) strongly agreed and 17 respondents (28%) agreed that restricting some antibiotics could prevent loss of antibiotic susceptibility.

### Antimicrobial stewardship

Although 44 (73%) of 60 respondents had heard of the term “antimicrobial stewardship,” 12 (27%) of these 44 did not know what it meant (Table 2). We detected a significant difference in knowledge of the term “antimicrobial stewardship” by staff role (*P* < .001). Nurses were less familiar with the term than pharmacists (*P* < .001) or physicians (*P* < .001).

**Fig. 2.**
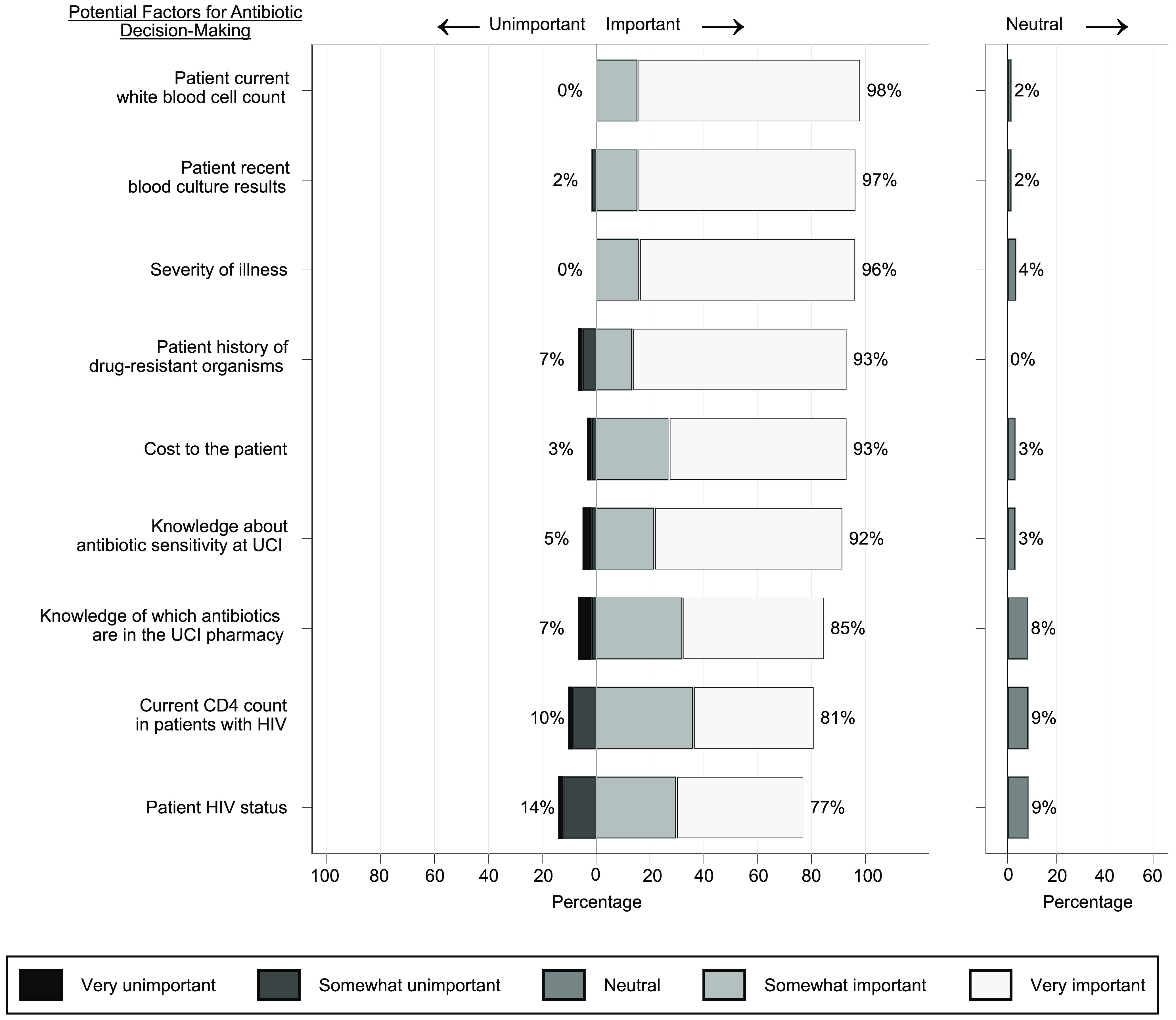
Factors that physicians, pharmacists, and nurses working at the Uganda Cancer Institute consider to be important when choosing antibiotics to treat infections. Percentages shown next to bars represent the combined total percentage of respondents reporting that the factor is somewhat or very unimportant (left of bars, main chart), somewhat or very important (right of bars, main chart), or neither important nor unimportant (right of neutral chart).

All but 1 respondent strongly agreed (52 of 60, 87%) or agreed (7of 60, 12%) that it would be good to have more guidance on antibiotic selection. We detected significant differences in the importance assigned to various factors when choosing antibiotics (*P* < .001). Patient white blood cell count, severity of illness, and recent blood culture results were considered significantly more important than either the patient’s HIV status or CD4 count (ie, T-cell test) (Fig. 2).

### Sources of antibiotic information and continuing antibiotic education

Of 61 respondents, 52 (85%) thought that knowledge about antibiotics was “very important” to their clinical profession. Almost two-thirds received training about antibiotics within the past year (Supplementary Table 1). Antibiotic training was less common among nurses than among pharmacists or physicians, although these differences were not statistically significant (*P* = .09). The most common training formats were teaching on patient rounds and in-person courses outside UCI (Supplementary Table 1).

**Fig. 3.**
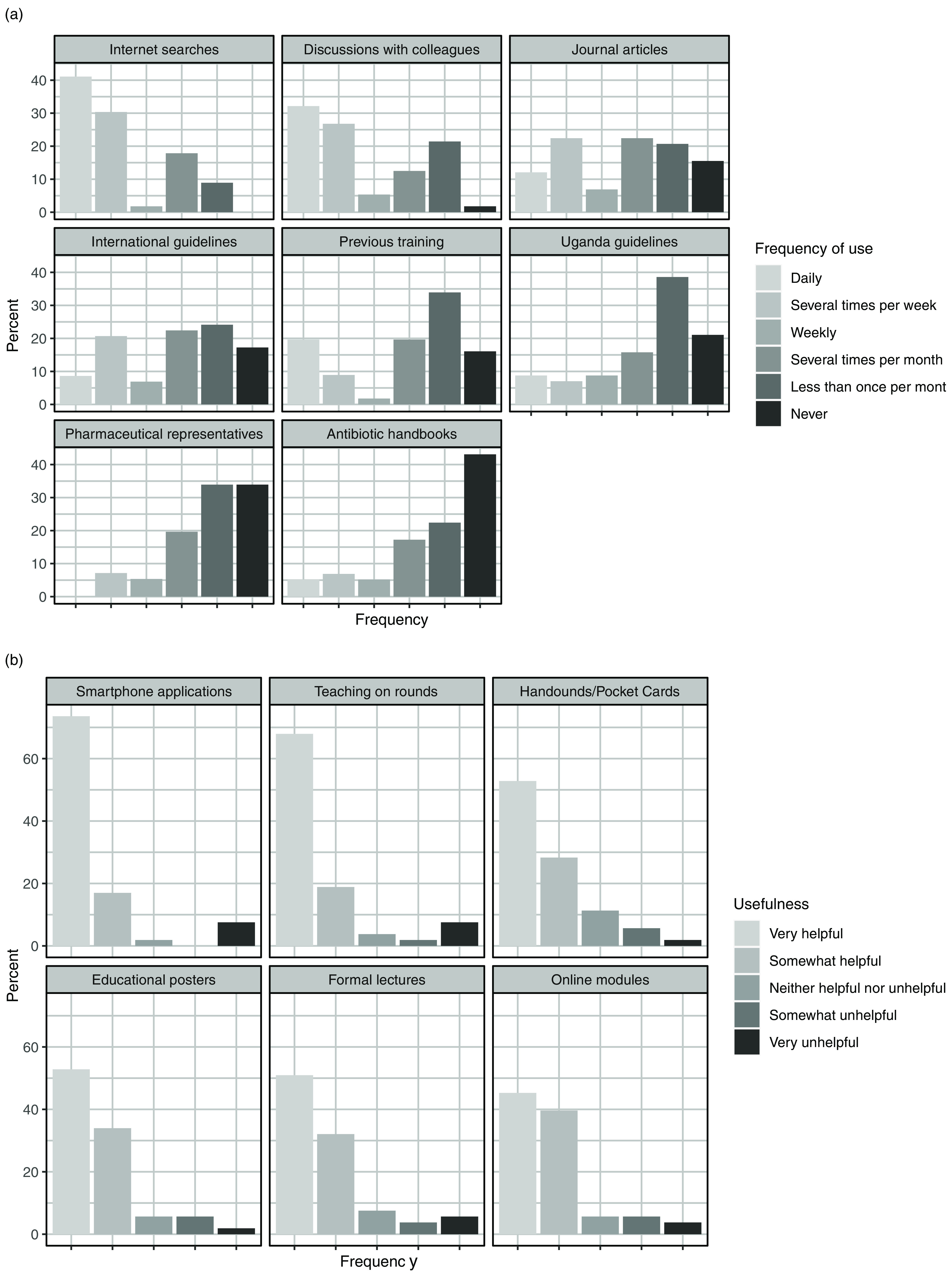
Sources of antibiotic information for physicians, nurses, and pharmacists working at the Uganda Cancer Institute. Figure 3A shows the current sources of information used when answering specific antibiotic questions. Figure 3B shows the educational formats felt to be useful for future antibiotic training. Results are arranged by median category for frequency of use (3A) or degree of usefulness (3B).

Among the 60 respondents, 39 reported using at least 1 source of information daily to answer antibiotic questions (Fig. 3a). We detected significant differences in the frequency with which respondents used these sources (*P* < .001); Internet searches were used significantly more frequently than any other source except for discussions with colleagues. Of 59 respondents, 56 (95%) agreed or strongly agreed that they would like to receive more training in antibiotic use. Figure 3b shows the formats that respondents felt would be most helpful for future antibiotic training.

### Diagnostic limitations

**Fig. 4.**
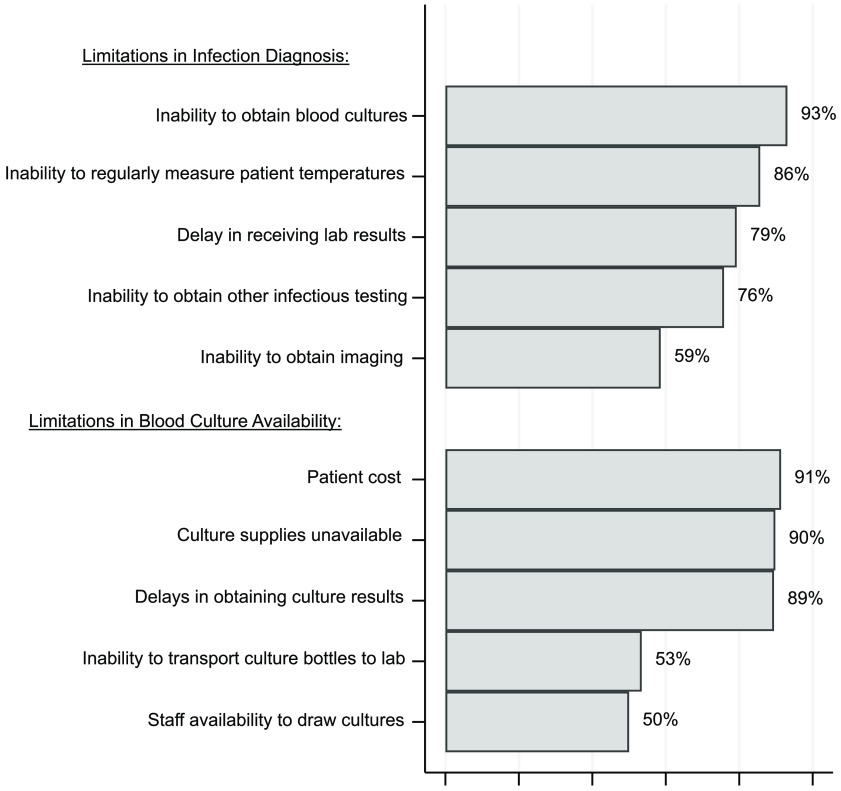
Factors that physicians, pharmacists, and nurses working at the Uganda Cancer Institute perceive as limiting the ability to diagnose infections and obtain blood cultures.

When asked about factors that limit infection diagnosis, inability to obtain blood cultures, inability to regularly measure patient temperatures, and delayed laboratory test results were most frequently identified (Fig. 4). Other factors identified included delays in seeing a physician, drug stockouts, and patient load. Respondents felt that cost, culture supply availability, and delayed culture results limited the ability to obtain and use blood cultures (Fig. 4).

### Antibiotic limitations

**Fig. 5.**
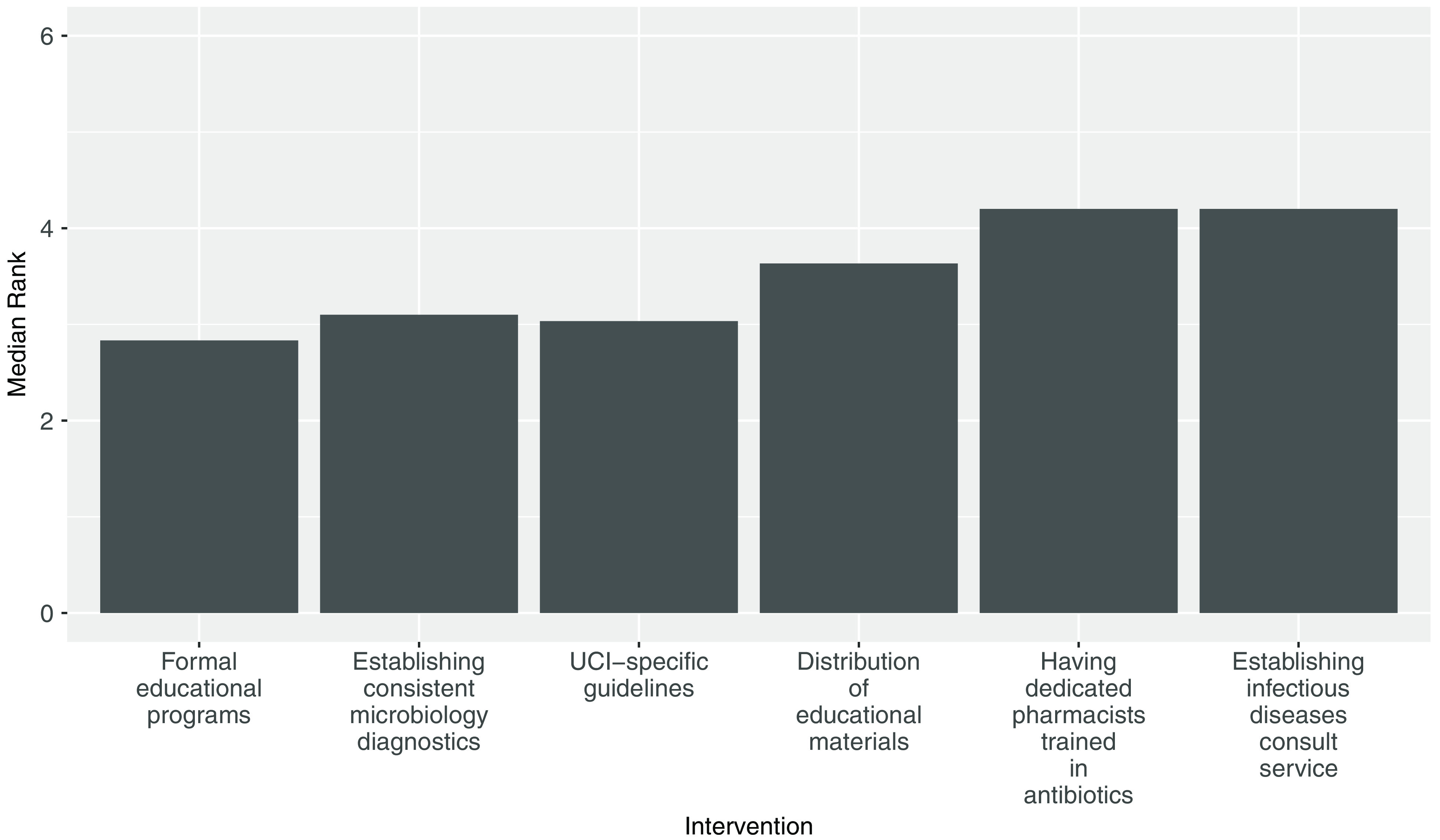
Interventions that nurses, pharmacists, and physicians perceive to be the most important to improving infection management at the Uganda Cancer Institute. Respondents ranked each intervention based on order of importance, with 1 being the most important and 6 the least important. Respondents who ranked all interventions were included (n = 30).

Of 61 respondents, 45 (75%) thought it was easy to know which antibiotics are available in the UCI pharmacy. Only 10 (16%) thought it was easy to find the appropriate antibiotics in the evenings, and only 22 (36%) thought it was easy to find the appropriate antibiotics on weekends. When asked to rank the interventions that could most improve infection management, disseminating educational materials, developing educational programs, and creating updated UCI-specific infection management guidelines were considered most important. (Fig. 5). Of 61 respondents, 40 (65%) suggested additional ways to improve infection diagnosis and management (Supplementary Table 2). Suggestions included improving early infection identification, providing blood cultures for all patients with suspected infections, creating a dedicated infection management team, and educating patients about proper antibiotic use.

## Discussion

In our cross-sectional survey, we evaluated the knowledge and attitudes regarding antimicrobial resistance and antimicrobial stewardship among nurses, pharmacists, and physicians working at a national cancer center in sub-Saharan Africa. Most respondents considered antibiotic knowledge very important to their clinical job and desired additional training in antibiotic use. We found that nurses were less familiar with the terms “antimicrobial resistance” and “antimicrobial stewardship” than pharmacists or physicians. Finally, we identified numerous barriers to diagnosing infections, including the ability to regularly monitor patient temperatures and to obtain blood cultures. These findings will inform our ongoing efforts to develop ASPs that account for the unique barriers encountered at UCI and in other cancer treatment programs throughout sub-Saharan Africa.

A primary goal of antimicrobial stewardship is to decrease the emergence, selection, and spread of antimicrobial resistance by optimizing antimicrobial use.^10,15^ To this end, it is important to educate clinicians about the causes of antimicrobial resistance.^16^ More than 80% of our survey respondents recognized antimicrobial resistance as a significant problem at UCI, which was higher than in previous surveys of healthcare workers from sub-Saharan Africa.^17,18^ This finding may reflect our ongoing efforts to educate staff about the high prevalence of MDR gram-negative bacterial infections at UCI.^2,19^ Among the UCI staff, there was general recognition that inappropriate antibiotic use contributes to antimicrobial resistance. Hand hygiene was not as well recognized as a contributing factor and represents an opportunity for improved education. Similar surveys from sub-Saharan Africa show that hand hygiene is an underrecognized contributor to resistance.^17,18,20^ Yet, good hand hygiene reduces transmission of resistant bacteria within the healthcare setting.^21^ For example, in a recent study from Mulago National Referral Hospital in Kampala, resistant gram-negative bacteria primarily spread through direct contact between patients and healthcare providers or nonmedical caretakers.^22^ It is also important to address environmental limitations that occur in low-resource settings (eg, lack of alcohol-based hand gel, inadequate handwashing facilities), which remain an ongoing challenge at UCI.

The appropriate use of microbiologic diagnostics to guide therapeutic decisions, or diagnostic stewardship, is another important aspect of antimicrobial stewardship.^23^ Diagnostic stewardship includes the ability to rapidly identify patients with suspected infections and to obtain the appropriate microbiologic laboratory tests. The ability to consistently monitor temperatures was seen as a key limitation to identifying patients with infections at UCI. In high-resource settings, temperatures are routinely measured 3–4 times daily and are, therefore, not included as a core component of diagnostic stewardship. However, in sub-Saharan Africa, 1 nurse may care for 30–70 patients,^24^ making it challenging to consistently measure vital signs. Developing strategies that account for staffing limitations could improve fever detection, facilitate blood culture collection, and decrease time to antibiotics. We are currently collaborating with UCI nurses to train patient family members to measure temperatures and alert nurses when a fever occurs. Since time from fever onset to the administration of guideline-recommended antibiotics is associated with outcomes,^25^ improved fever detection could decrease mortality for patients receiving cancer treatment in sub-Saharan Africa.

Obtaining blood cultures for patients with suspected infections is also an important component of diagnostic stewardship. Blood cultures decrease the use of unnecessary broad-spectrum antibiotics by allowing clinicians to tailor a patient’s antibiotic therapy to the causative organisms.^10,23,25^ They are also critical for developing a hospital antibiogram, in which the antimicrobial susceptibilities of organisms isolated at that hospital are aggregated into easily referenced tables.^10,23,26^ The antibiogram allows clinicians to track changes in antibiotic susceptibility and adapt evidence-based institutional treatment guidelines accordingly.^27^ In our study, patient cost was the most frequently identified barrier to obtaining blood cultures, which is consistent with findings from previous studies in sub-Saharan Africa.^28^ In Uganda, blood cultures cost up to $10.00 USD (ie, 4–5 days’ average wages),^29^ which is prohibitively expensive. Since blood cultures are essential for diagnosing and treating infections in patients with cancer, policy makers should consider these a core component of cancer care and factor these into the cost of cancer treatment.

To improve rational antibiotic decision making, it is necessary to understand provider prescribing practices. Among UCI clinicians, patient white blood cell count was most frequently considered to be “very important” when choosing antibiotics. This is fitting because patients with neutropenic fever are at high risk of infection-related complications.^11^ Fewer respondents identified HIV status or CD4 count as important. However, there is evidence that patients with HIV are at higher risk of developing treatment-related infections than their HIV negative counterparts.^30^ Those with HIV have high rates of antibiotic exposure^31,32^ and are at increased risk of developing infections with resistant bacteria.^32,33^ The microbiology of febrile illness also varies by HIV status. For example, in sub-Saharan Africa tuberculosis is a leading cause of sepsis among those with HIV.^34^ Because one-third of patients with cancer in sub-Saharan Africa also have HIV,^35^ understanding the relationship between HIV status and treatment-related infections will inform locally relevant guidelines for cancer-related infections.

Educational interventions improve healthcare workers’ ability to prescribe the appropriate antibiotics.^10^ Almost two-thirds of our respondents received antibiotic teaching within the past year. Most received teaching during clinical care activities and used Internet searches and discussions with colleagues to answer antibiotic questions, indicating that just-in-time learning was a primary educational strategy. Respondents also considered readily accessible materials such as smartphone applications, teaching on ward rounds, and informational handouts to be “very helpful” learning formats. Although continuing medical education is traditionally delivered through didactic lectures,^36^ active learning strategies (eg, audit and feedback, case-based learning) and multimodal interventions increase knowledge retention and translation into clinical practice. The COVID-19 pandemic highlights the importance of using multimodal strategies, particularly for those living in low-resource settings. Online learning platforms are expensive and challenging to use when there is inconsistent Internet access. On-demand mobile tools (eg, smartphone apps) increase access to critical information during routine patient care and improve knowledge sharing across educational sites.^37^ Incorporating these tools into our ASP could improve evidence-based infectious disease management at our center.

Overall, in our study, nurses were less familiar with the terms “antimicrobial resistance” and “antimicrobial stewardship” than physicians or pharmacists. At UCI and in other hospitals in low-resource settings, nurses are often responsible for identifying patients with infections and initiating antibiotics. Thus, it is critical for oncology nurses to understand how to select the appropriate antibiotics.^24^ Most stewardship interventions target physicians and pharmacists; thus, nurses are underutilized members of the stewardship team. In high-resource settings, nurse-driven protocols have been developed to facilitate blood culture collection and antibiotic initiation for patients with neutropenic fever.^38^ In sub-Saharan Africa, similar protocols have been developed to manage bacterial meningitis.^39^ These protocols improve guideline adherence, decrease time-to-antibiotics, and improve patient outcomes. Developing nurse-led protocols and training nurses in the principles of antimicrobial stewardship are ways to adapt ASPs for patients receiving cancer treatment in low-resource settings.

Our study had several limitations. This survey was completed at a single cancer center in sub-Saharan Africa, and the results may not be representative of other cancer centers. Given the numerous infectious diseases initiatives underway at UCI, knowledge of antimicrobial resistance and antimicrobial stewardship may be higher at UCI than in other cancer treatment programs. Those who responded may also have more training or inherent interest in the topic. In addition, some respondents did not answer every question, which influenced our ability to compare answers across questions. Because the survey took place during the COVID-19 pandemic, the frequency of education, concerns about supply availability, and issues with antibiotic shortages may not be typical. However, studies completed in low-resource settings before the COVID-19 pandemic had similar findings regarding antimicrobial education and antibiotic supply availability.^18,40^

In this survey, we evaluated the knowledge and perceptions of antimicrobial resistance and stewardship among physicians, nurses, and pharmacists working at a single cancer hospital in sub-Saharan Africa. UCI staff were knowledgeable about the term antimicrobial resistance and eager to learn more about antibiotic use. We also identified several areas for targeted interventions. These include increasing education regarding the importance of hand hygiene and appropriate antibiotic selection, allocating resources for blood cultures, implementing strategies to improve fever detection, and incorporating nurses as key members of the antimicrobial stewardship team. Our findings will inform the development of ASPs for cancer treatment programs in low-resource settings.

## Supplementary material

For supplementary material accompanying this paper visit https://doi.org/10.1017/ash.2022.28. click here to view supplementary material
