# Supplementary material for: Knowledge and perceptions of antimicrobial resistance and antimicrobial stewardship among staff at a national cancer referral center in Uganda
Source: Antimicrob Steward Healthc Epidemiol. 2022 Apr 6;2(1):e54. doi: 10.1017/ash.2022.28 (PMC9726558; doi:10.1017/ash.2022.28)
Supplement: Supplementary file 1 [file ashsup.zip › S2732494X22000286sup001.docx]

| **Supplemental Table 2.** Respondent suggested interventions to improve infection diagnosis and management at the Uganda Cancer Institute.^a^ | | |
| --- | --- | --- |
| **Category** |  | **Intervention** |
| Guidelines | Guideline development | Develop recommendations for early infection detection**^b^** |
|  |  | Develop recommendations for empiric antibiotic use**^b^** |
|  |  | Ensure that guidelines are usable for all healthcare team members (e.g., nurses, doctors) |
|  | Guideline dissemination | Guidelines disseminated to all medical staff**^b^** |
|  |  | Paper copy of guidelines available on each ward |
|  |  | Electronic version of guidelines available via smartphone application |
| Education | Healthcare workers | Hold regular continuing medical education courses about appropriate antibiotic use**^c^** |
|  |  | Regular updates on new antibiotics and available treatments |
|  |  | More research done regarding resistant organisms |
|  | Patients/  family-members | Teach patients and their families how to recognize the signs and symptoms of infections**^b^** |
|  |  | Teach patients and their families how to correctly use the prescribed antibiotics**^c^** |
| Infection prevention | Systems-level | Develop infection control and prevention measures for the inpatient wards**^b^** |
|  |  | Proper use of personal protective equipment |
|  | Patient-level | Early initiation of infection prophylaxis |
| Diagnostic Testing | Laboratory services | Laboratory services available 24/7**^b^** |
|  |  | Microbiology services available at on-site laboratory**^b^** |
|  | Blood cultures | Blood cultures provided as standard of care for all patients with suspected infections **^c^** |
|  |  | Blood cultures collected before antibiotics are started**^b^** |
|  |  | Laboratory results returned to the clinical team in a timely manner**^b^** |
|  | Radiology services | Ability to rapidly obtain imaging |
| Pharmacy | Available Services | Longer pharmacy hours (e.g., open longer in evenings/weekends) **^b^** |
|  |  | Specialty trained infectious diseases pharmacist on staff |
|  | Available antibiotics | Develop the antibiotic formulary based on local culture sensitivity results **^b^** |
|  |  | Ensure formulary antibiotics are stocked and available for patients**^c^** |
|  |  | Procurement of high-quality antibiotics |
|  |  | Antibiotics properly stored |
| Clinical management | Clinical team structure | Create a dedicated team to care for patients with infections**^c^** |
|  |  | Develop treatment plans collaboratively with the multidisciplinary team |
|  |  | Create a handoff process for teams caring for patients with infections |
|  | Fever detection | Vital signs routinely done**^b^** |
|  |  | Early identification of patients with infections**^c^** |
|  |  | Doctors assess patients more rapidly after fever is identified |
|  | Antibiotic selection and use | Rapid antibiotic initiation |
|  |  | Antibiotics prescribed based on culture and sensitivity |
|  |  | Routinely monitor patients for their clinical response to antibiotics |
| **^a^** Based on responses to the open-ended question “Do you have any additional thoughts about how we can improve the diagnosis and management of patients with infections at the Uganda Cancer Institute?” In total, 40 respondents (24 nurses, 5 pharmacists, and 11 doctors) answered the question.  **^b^** Interventions suggested by at least 2 respondents  **^c^** Interventions suggested by 5 or more respondents | | |
