# Supplementary material for: Knowledge and perceptions of antimicrobial resistance and antimicrobial stewardship among staff at a national cancer referral center in Uganda
Source: Antimicrob Steward Healthc Epidemiol. 2022 Apr 6;2(1):e54. doi: 10.1017/ash.2022.28 (PMC9726558; doi:10.1017/ash.2022.28)
Supplement: Supplementary file 1 [file ashsup.zip › S2732494X22000286sup002.docx]

| **Supplemental Table 1.** Types of antibiotic education UCI doctors, nurses, and pharmacists received within the past year. | | | | | |  |
| --- | --- | --- | --- | --- | --- | --- |
|  | **n^b^** | **All** | **Nurses** | **Pharmacists** | **Physicians** | |
| Number who received education^a^ | 61 | 38 (62) | 14 (48) | 6 (86) | 18 (72) | |
| **Educational formats used by those who received teaching (n=38)** | | | | | |  |
| Teaching on patient rounds or during clinical care activities | 36 | 27 (75) | 9 (69) | 2 (33) | 16 (94) | |
| In-person medical courses or medical training sessions outside of UCI | 35 | 24 (69) | 10 (77) | 4 (67) | 10 (62) | |
| Online medical course or medical training | 33 | 13 (39) | 6 (46) | 2 (40) | 5 (33) | |
| In-person conferences or lectures at UCI | 33 | 12 (36) | 2 (17) | 1 (20) | 9 (56) | |
| _­­­_Data are presented as No. (%).  ^a^Number of survey respondents who answered each question. | | | | | |  |
